# Prediction of COVID-19 Hospital Length of Stay and Risk of Death Using Artificial Intelligence-Based Modeling

**DOI:** 10.3389/fmed.2021.592336

**Published:** 2021-05-04

**Authors:** Bassam Mahboub, Mohammad T. Al Bataineh, Hussam Alshraideh, Rifat Hamoudi, Laila Salameh, Abdulrahim Shamayleh

**Affiliations:** ^1^Clinical Sciences Department, College of Medicine, University of Sharjah, Sharjah, United Arab Emirates; ^2^Sharjah Institute for Medical Research, University of Sharjah, Sharjah, United Arab Emirates; ^3^Industrial Engineering Department, American University of Sharjah, Sharjah, United Arab Emirates; ^4^Industrial Engineering Department, Jordan University of Science and Technology, Irbid, Jordan; ^5^Division of Surgery and Interventional Science, University College London, London, United Kingdom

**Keywords:** artificial intelligence, COVID-19, length of stay, predictive analytics, risk of death

## Abstract

Severe acute respiratory syndrome coronavirus 2 (SARS-CoV-2) is a highly infectious virus with overwhelming demand on healthcare systems, which require advanced predictive analytics to strategize COVID-19 management in a more effective and efficient manner. We analyzed clinical data of 2017 COVID-19 cases reported in the Dubai health authority and developed predictive models to predict the patient's length of hospital stay and risk of death. A decision tree (DT) model to predict COVID-19 length of stay was developed based on patient clinical information. The model showed very good performance with a coefficient of determination *R*^2^ of 49.8% and a median absolute deviation of 2.85 days. Furthermore, another DT-based model was constructed to predict COVID-19 risk of death. The model showed excellent performance with sensitivity and specificity of 96.5 and 87.8%, respectively, and overall prediction accuracy of 96%. Further validation using unsupervised learning methods showed similar separation patterns, and a receiver operator characteristic approach suggested stable and robust DT model performance. The results show that a high risk of death of 78.2% is indicated for intubated COVID-19 patients who have not used anticoagulant medications. Fortunately, intubated patients who are using anticoagulant and dexamethasone medications with an international normalized ratio of <1.69 have zero risk of death from COVID-19. In conclusion, we constructed artificial intelligence–based models to accurately predict the length of hospital stay and risk of death in COVID-19 cases. These smart models will arm physicians on the front line to enhance management strategies to save lives.

## Introduction

The current century has witnessed several emerging pandemics, such as severe acute respiratory syndrome (SARS), Middle East respiratory syndrome (MERS), Ebola, and Zika viruses. Since December 2019, the world has been impacted by the spread of the coronavirus SARS-CoV2. The coronavirus disease 2019 is an infectious disease that causes severe acute respiratory illness. In March 2020, the World Health Organization (WHO) characterized the COVID-19 outbreak as a pandemic due to the alarming levels of spread and severity. The new virus has strong human-to-human transmission and can cause severe pneumonia to death. The fast spread of COVID-19 posed a significant challenge to healthcare systems and specifically to hospitals due to the surge in caseload per hospital (1). The challenges were through different stages, including testing patients who were potentially infected, treating patients who are infected, and preparing to treat future patients. Therefore, there is a vital need to precisely predict and triage cases and rank those with high probability to progress to a higher level of illness severity.

Infection control can reduce disease incidences and attributed morbidity and mortality, such as cardiovascular and respiratory complications (2). Vaccine and drug development is a lengthy process that requires many clinical trials before being proven safe and effective; therefore, early detection and prevention is the best approach to control pandemics by blocking transmission. Among the current analytical tools that can support infection control is artificial intelligence (AI) (3). AI has been widely recognized as one of the most powerful and promising analytical tools for humankind, utilizing big data input to identify complex patterns based on data structure after filtration and integration. Most health-related challenges require the support of bioinformaticians and statisticians to resolve big data complexities and provide optimized healthcare decisions. For example, a research team from the Mayo Clinic optimized clinical ICU settings using an AI-based application called ambient warning and response evaluation, which resulted in timely and accurate decision making while reducing the length of stay (LOS) by 37% (4). Another research group from University College London developed a machine learning model that predicts risk of death in coronary artery disease patients more accurately than clinical experts (5).

A recent AI-based report demonstrates an initial phase model to predict which COVID-19 patients will develop severe respiratory disease, and although the study was limited to the size of the data set, it successfully showed 70–80% successful prediction (6). Other studies apply AI to determine the important indices regarding COVID-19 diagnosis and tracking systems to improve clinical outcomes (7, 8).

In this study, we aim to use multivariate analysis and a decision tree (DT) algorithm to build a predictive model that accurately predicts COVID-19 subject's LOS and risk of death based on demographic and clinical attributes at hospital admission in the United Arab Emirates.

## Methods

### Ethical Consideration

The study protocol was reviewed and approved by Dubai Scientific Research Ethical Committee (DSREC), Dubai Health Authority (DHA); the ethical approval number of the study is DSREC-04/2020_11.

### Study Population

In this retrospective study, we analyzed clinical records of 2017 confirmed COVID-19 patients admitted for treatment at Rashid Hospital, DHA in Dubai between January 1, 2020, and July 20, 2020. SARS-CoV-2 infection was confirmed by real-time reverse-transcription polymerase-chain-reaction (RT-PCR) assay from nasal and pharyngeal swab specimens according to their ICD10 disease code for all patients enrolled in this study. Patient records were fully anonymized prior to access to attain patient's privacy.

### Sample Size

The main objective of this study is to report the proportion of patients with certain disease characteristics and to compare those proportions among COVID-19 disease severity levels. Assuming a total number of COVID-19 confirmed cases at the time of study of 58,249 cases in the UAE, a 5% margin of error, and a 95% confidence level, a sample of a minimum size of 382 patients is needed.

### Data Description and Pre-processing

Patients' records, including clinical and non-clinical data were extracted and used in this study. Non-clinical data included age, gender, nationality, body mass index, and travel history. Clinical patient data included three data categories: diagnosis-related data, medication-related data, and laboratory-related data. Diagnosis-related attributes included are principal diagnosis, admission unit, level of care at admission, and medical history and comorbidities. All medications administered throughout the patient stay at the hospital were extracted from the hospital database and then were categorized according to their main functionality. Laboratory data included the results of all blood tests performed at patient admission. The latest available laboratory tests included are white blood cells (WBCs), blood hemoglobin, platelet count (PLT), lymphocyte count, sodium (Na+), potassium (K+), urea, creatinine, alkaline phosphatase (ALP), aspartate transaminase (AST), alanine aminotransferase (ALT), bilirubin, international normalized ratio (INR), lactate dehydrogenase (LDH), glucose-6-phosphate dehydrogenase (G6PD), procalcitonin (PCT), C-reactive protein (CRP), ferritin, hemoglobin A1c (HbA1c), dimer, peptides B-type natriuretic peptide (BNP), atrial natriuretic peptide (ANP) (Pro-PNP), creatine kinase-MB (CK-MB), and vitamin D. To assess patient health status and identify the required level of care, the hospital follows the modified early warning score (MEWS) health assessment system 10. The MEWS system uses physiological parameters, such as blood pressure; heart rate; respiratory rate; temperature; and alert, verbal, pain, unresponsive (AVPU) neurological score to assign a score for each patient. The higher the score, the more severe is the patient's health status and the more the required patient care. Based on MEWS scores, patients were categorized into three levels according to their COVID-19 disease severity. Patients with a MEWS score of <2 were considered at the mild disease severity level. Patients with a MEWS score in the range 3–5 were considered at the moderate severity level, and patients with a MEWS score of six or more were considered at the severe level. MEWS score changes on a daily basis depending on the patient's clinical status, and the highest achieved score throughout the patient stay at the hospital was used for the severity assessment. Descriptive statistics, including means, frequencies, and proportions, are summarized for the collected data. Summaries are stratified by the disease severity level. Chi-squared and Fisher exact-tests were used whenever appropriate to examine differences among categorical predictors. A significance level of *p* < 0.05 is used throughout the study.

### Predictive Modeling

To help healthcare facilities in resource planning, we provide two AI-based prediction models to better assess healthcare demand. Patients' hospital LOS is predicted in the first model, and the patient's risk of death as a severe disease consequence is predicted by the second model. We constructed two prediction models instead of single model with two outcomes due to the heterogeneous input attributes required for each model and to provide better model application flexibility so that hospitals can adopt the model of interest. Available clinical and non-clinical patient information from 2017 patients at initial evaluation were used as inputs for both models. Models were trained on 75% of patients' records, and the remaining 25% were used for performance evaluation of the models. At the training phase, model performance was tuned and validated through a tenfold cross-validation technique (9). The conditional recursive partitioning tree algorithm known as ctree was used for model building (10). The algorithm allows for missing patient data through the use of surrogate attributes. For predicting LOS, patients with missing LOS values because they were still hospitalized were dropped out. Unsupervised hierarchical clustering was carried out using a Euclidean distance measure and Ward linkage. All data analysis and model building tasks were done using R (version 3.6) statistical computing software (11).

## Results

### COVID-19 Patient Characteristics

In this study, a retrospective analysis of medical records for 2017 COVID-19-positive patients who were evaluated between January 1, 2020, and July 20, 2020, at Rashid Hospital was conducted, and 1,770 (87.8%) of those patients were males and 247 (12.2%) were females. The percentage of patients reporting traveling outside the UAE was <5%. The overall mean age was 43.9 years with a standard deviation of 12.4 years. The majority of the patients were Asians (81.0%) and from the MENA region (13.9%). According to the patients' BMI, about 64.1% of them were overweight or obese. Furthermore, 93.64% of the patients had a positive rhesus (Rh) reagent blood group. A summary of patient characteristics based on their disease severity is shown in Table 1. The summary in Table 1 shows a clear positive relationship between age and disease severity. Higher values of patient age are associated with more serious disease cases. Similarly, higher levels of BMI are associated with higher disease severity.

**Table 1:** Summary of COVID-19 patients' characteristics by required level of care at admission.

	**Mild**	**Moderate**	**Severe**	**Overall**
	**(*N* = 849)**	**(*N* = 628)**	**(*N* = 540)**	**(*N* = 2,017)**
**Age**
Mean (SD)	42.2 (12.5)	43.5 (11.7)	47.1 (12.3)	43.9 (12.4)
**Gender**
Female	145 (17.1%)	44 (7.01%)	58 (10.7%)	247 (12.2%)
Male	704 (82.9%)	584 (93.0%)	482 (89.3%)	1,770 (87.8%)
**Nationality**
African	28 (3.31%)	13 (2.07%)	17 (3.18%)	58 (2.89%)
Asian	634 (74.9%)	546 (86.9%)	447 (83.6%)	1,627 (81.0%)
MENA	153 (18.1%)	64 (10.2%)	62 (11.6%)	279 (13.9%)
Western	31 (3.66%)	5 (0.796%)	9 (1.68%)	45 (2.24%)
Missing	3 (0.4%)	0 (0%)	5 (0.9%)	8 (0.4%)
**Blood group**
A Negative	7 (1.96%)	3 (0.789%)	4 (1.48%)	14 (1.39%)
A Positive	97 (27.2%)	88 (23.2%)	61 (22.5%)	246 (24.4%)
AB Negative	1 (0.280%)	3 (0.789%)	2 (0.738%)	6 (0.595%)
AB Positive	16 (4.48%)	32 (8.42%)	22 (8.12%)	70 (6.94%)
B Negative	11 (3.08%)	4 (1.05%)	6 (2.21%)	21 (2.08%)
B Positive	88 (24.6%)	104 (27.4%)	85 (31.4%)	277 (27.5%)
O Negative	12 (3.36%)	6 (1.58%)	5 (1.85%)	23 (2.28%)
O Positive	125 (35.0%)	140 (36.8%)	86 (31.7%)	351 (34.8%)
Missing	492 (58.0%)	248 (39.5%)	269 (49.8%)	1,009 (50.0%)
**BMI**
Underweight	20 (2.39%)	8 (1.29%)	7 (1.31%)	35 (1.75%)
Normal	312 (37.2%)	207 (33.3%)	163 (30.4%)	682 (34.2%)
Overweight	341 (40.7%)	275 (44.2%)	236 (44.0%)	852 (42.7%)
Obese	165 (19.7%)	132 (21.2%)	130 (24.3%)	427 (21.4%)
Missing	11 (1.3%)	6 (1.0%)	4 (0.7%)	21 (1.0%)

### Analysis of COVID-19-Related Sign- and Symptom-Based Diagnosis at Initial Evaluation

The list of health problems as diagnosed by the physicians at admission was categorized according to the following: respiratory, endocrinology, CVS, renal, CNS, and GIT, among others. The percentages of the presence of those health problems among admitted patients are summarized in Supplementary Figure 1, according to COVID-19 disease severity. Results in Supplementary Figure 1A indicate that about 12.3% of the admitted patients had CVS health problems and 4.6% of them had GIT health problems. Reported *p*-values indicate a significantly different distribution of presence in renal and CNS health problems across the levels of disease severity.

As COVID-19 is a respiratory system–related health problem, almost all patients across all levels of disease severity had their main diagnoses as a respiratory-related problem (Supplementary Figure 1A). Two main respiratory-related problems were of at most importance for the physicians to identify the disease severity, namely pneumonia and upper respiratory tract Infection (URTI). About 52.8 and 10.2% of patients had pneumonia and URTI, respectively (Supplementary Figure 1B). Reported *p*-values indicate significantly different presence levels of these two problems across disease severity levels. Pneumonia had the highest presence at the moderate (66.6%) and severe (62.6%) COVID-19 severity levels, and URTI had the highest presence at the mild (13.4%) severity level.

### Laboratory Tests Result of COVID-19 Patients at Initial Evaluation

**Figure 1:**
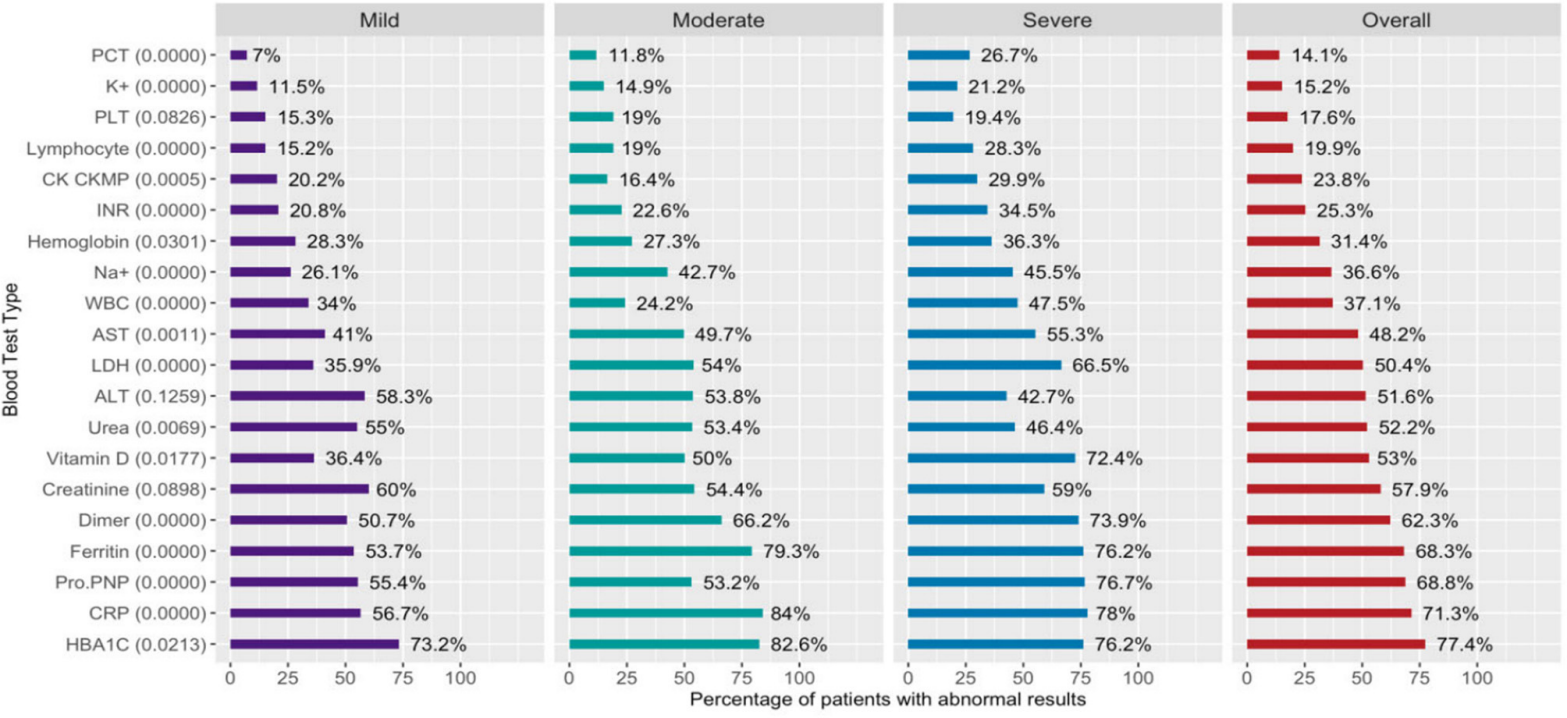
Percentage of patients with abnormal laboratory test results. Percentage of patients with abnormal blood test results was calculated as the proportion of patients with blood test results outside the normal range at each disease severity level. Numbers in parentheses are the Chi-squared *p*-values for testing for significant differences among severity levels.

Percentages of patients with abnormal lab test results are shown in Figure 1 stratified by disease severity level. Percentages shown in Figure 1 indicate that abnormal test results are highly probable among patients with severe SARS-CoV2 infection levels. Overall, the top three abnormal lab test results were HBA1C (77.4%), CRP (71.3%), and Pro.PNP (68.8%). Patients had the lowest abnormal results in PCT and potassium (K+), but they were more common among severe-level patients (26.7 and 21.2%, respectively). To better understand the relationship between lab test results and disease severity, an unsupervised clustering of patients heat map was constructed and is shown in Supplementary Figure 2 using Euclidean distance measure and Ward linkage. The heat map in Supplementary Figure 2 indicates different distributions of Na+, lymphocyte count, INR, dimer, and CRP among the disease severity levels. Vitamin D, Pro.PNP, PCT, and AST results were similar across all levels of severity, confirming the AI models' validity.

### Evaluation of Prescribed COVID-19 Medications

Medications prescribed to all admitted patients during their treatment management course were categorized according to their basic functionality to the categories shown in Supplementary Figure 3. The summary in Supplementary Figure 3 indicates the top three medications are painkillers (73.5%), antiviral (51.7%), and antimicrobial (46.7%). Generally, patients at the mild severity level had the lowest prescribed drug percentages among almost all medication types.

**Figure 2:**
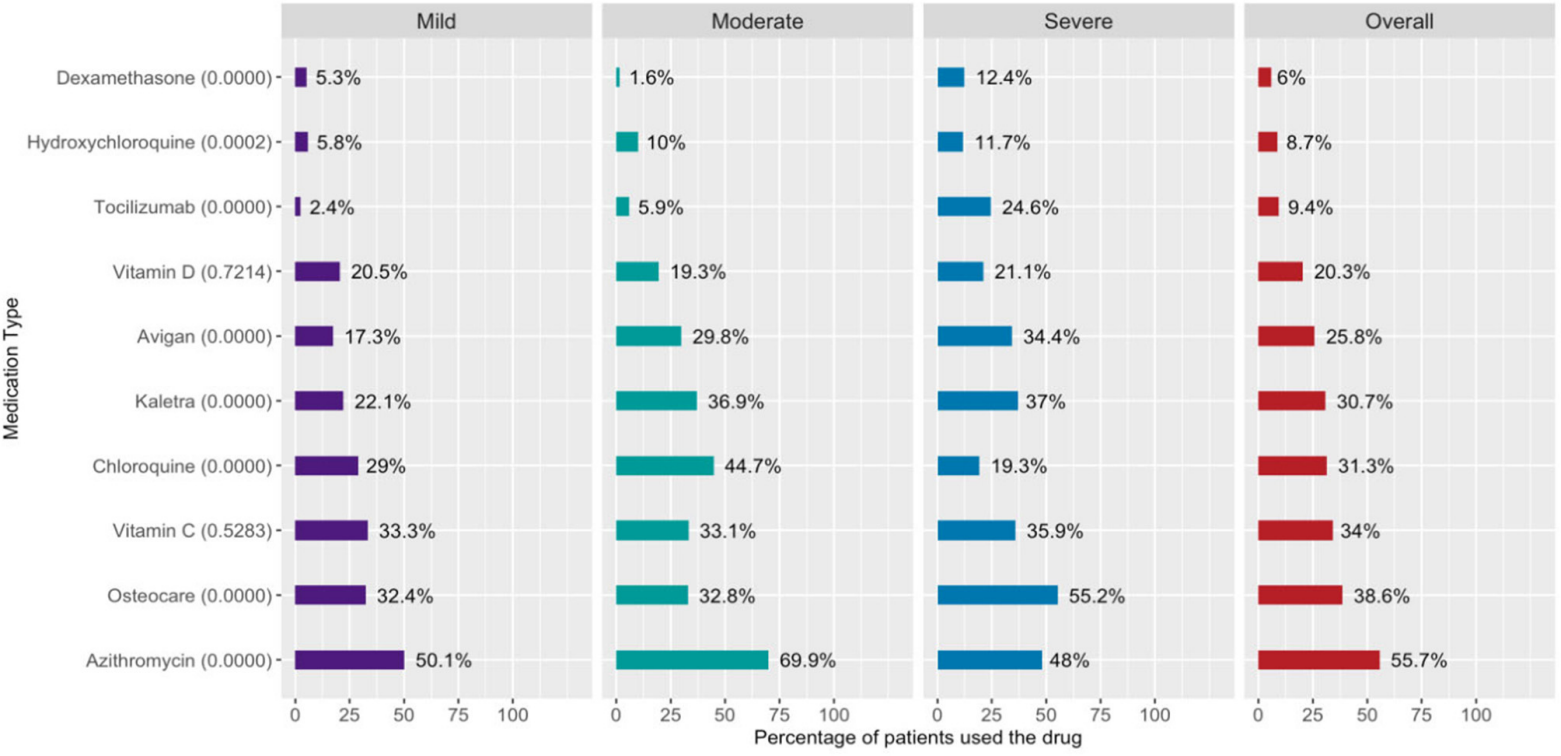
Percentage of patients prescribed COVID-19 treatment drugs. Percentage of patients prescribed COVID-19 medications shown in the *y*-axis of the figure. Proportions were calculated as the proportion of patients prescribed the medication of all patients at that severity level. Numbers in parentheses are the Chi-squared *p*-values for testing for significant differences among severity levels.

According to the hospital's COVID-19 treatment protocol, approved COVID-19 medications and the percentages of patients prescribed those medications are shown in Figure 2.

The results in Figure 2 show that azithromycin (55.7%) was the highest prescribed drug followed by Osteocare (38.6%), vitamin C (34.0%), chloroquine (31.3%), and lopinavir/ritonavir (Kaletra) (30.7%). Last, dexamethasone was the lowest prescribed drug with about 6% overall use.

### Predicting COVID-19 Patients' Hospital LOS at Initial Evaluation

**Figure 3:**
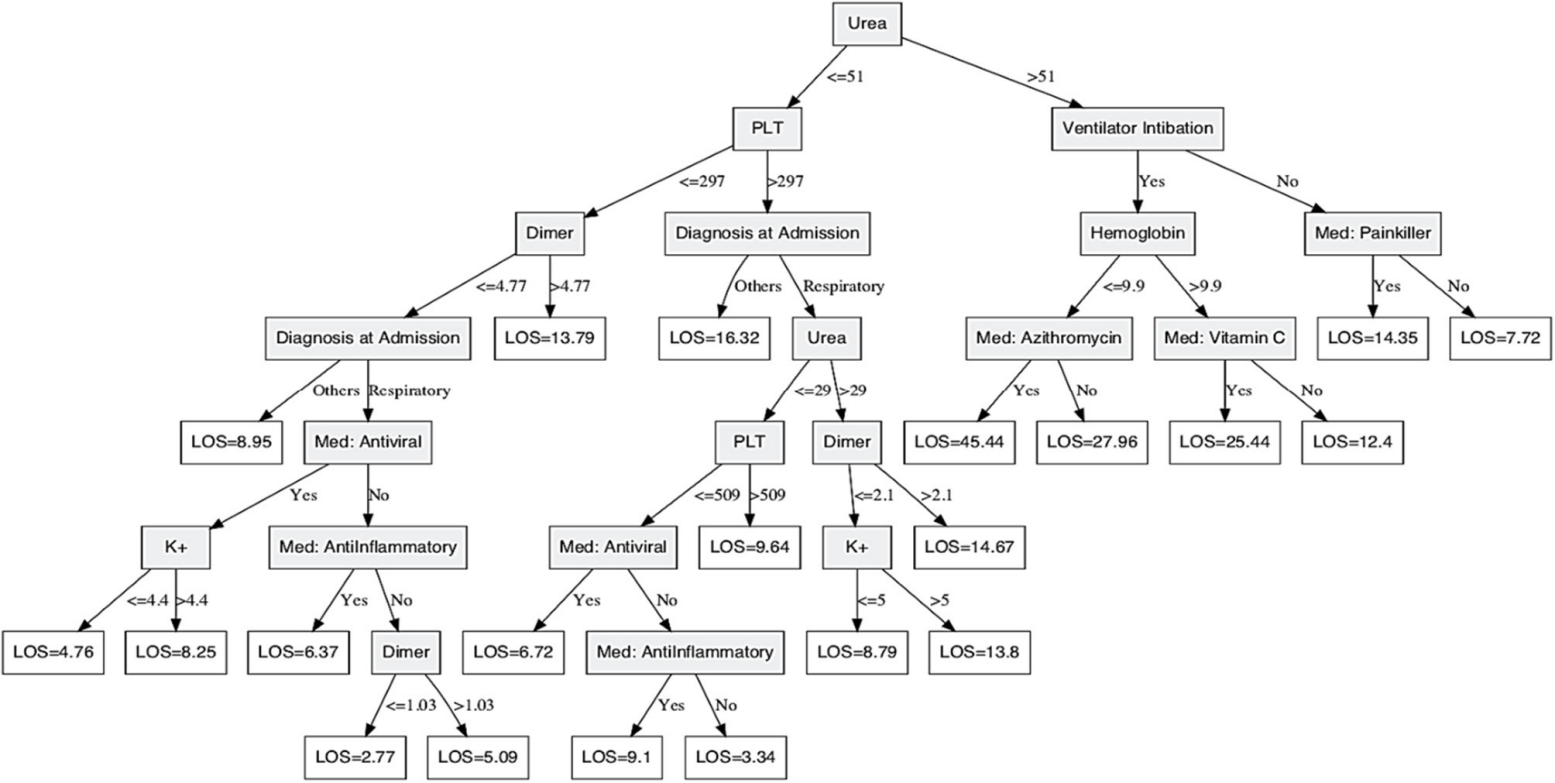
Decision Tree model for predicting COVID-19 patients' hospital length of stay. A secision tree prediction model was constructed to predict patient's hospital length of stay given demographic and clinical attributes at initial evaluation. Shaded nodes are the parent/split nodes, and the non-shaded nodes are the leaf nodes of the DT model. The conditional recursive partitioning tree algorithm (ctree) was used for model building.

From a management point of view, it is of great importance to know in advance how long each patient will stay in the hospital, known as the hospital LOS, to better allocate resources. A DT prediction model was constructed to predict the patient's LOS given the demographic and clinical attributes at initial evaluation. The obtained DT model is shown in Figure 3 for predicting LOS measured in days of stay at the hospital. Patients' data were divided into 75% for training and 25% for testing. The model was built and validated through the training part of the data, and the testing part was used to assess the constructed model performance. Available patient information, including demographics, list of health problems, diagnosis, medications administered, and lab test results, were used as model inputs. The model shows very good performance with a coefficient of determination *R*^2^ of 49.8%, a mean absolute deviation (MAD) of 3.9 days, and median absolute deviation of 2.85 days. Based on the obtained DT model, diagnosis at admission; use of ventilator intubation; medications: painkillers, azithromycin, antiviral, anti-inflammatory, and vitamin C; and lab tests: urea, PLT, dimer, K+, and hemoglobin were the significant attributes for predicting LOS. For example, COVID-19 patients with urea levels more than 51 mg/dL and not on ventilation stay for 7.72 days on average in the hospital if they do not use painkillers although their stay could be as long as 14.35 days if they use painkillers. Similarly, patients with urea levels of more than 51 mg/dL, intubated, and their hemoglobin levels more than 9.9 g/dL stay for 12.4 days, on average, in the hospital if they do not use vitamin C although their stay is about 25.44 days on average if they use vitamin C. However, patients with urea levels <51 mg/dL, PLT level <297 thousand platelets/μL, dimer level <1.03 ug/ml FEU, have no comorbidities, and have not used antiviral or anti-inflammatory medications stay in the hospital for 2.77 days on average.

DT models are known for their simple-to-interpret and easy-to-apply structure, which makes them the most common prediction tool in the medical field. Unfortunately, they are sensitive to outliers in the data, and therefore, it is important to test their robustness. To do so, variable importance estimated through the random forest ensembling method was estimated and is shown in Supplementary Figure 4 (12).

Results in Supplementary Figure 4 indicate that, by far, the most important variable in predicting patients' LOS is the use of ventilators, followed by hemoglobin and anti-inflammatory drug usage. Variables at the lower part of Supplementary Figure 4 indicate similar importance, and hence, their contribution to model performance is similar. The provided DT model in Figure 3 has picked up the top three most important variables for predicting LOS. Other variables in the model are chosen by the algorithm to improve the prediction accuracy, and all were of the top 30 important variables as shown in Figure 3. Furthermore, the same DT algorithm was used to train a DT model assuming the availability of different subsets of the original data. Comparisons of obtained models show that the selected attributes and their cut points were very similar across all model variations, indicating a robust DT model.

### Predicting COVID-19 Patient Risk of Death

**Figure 4:**
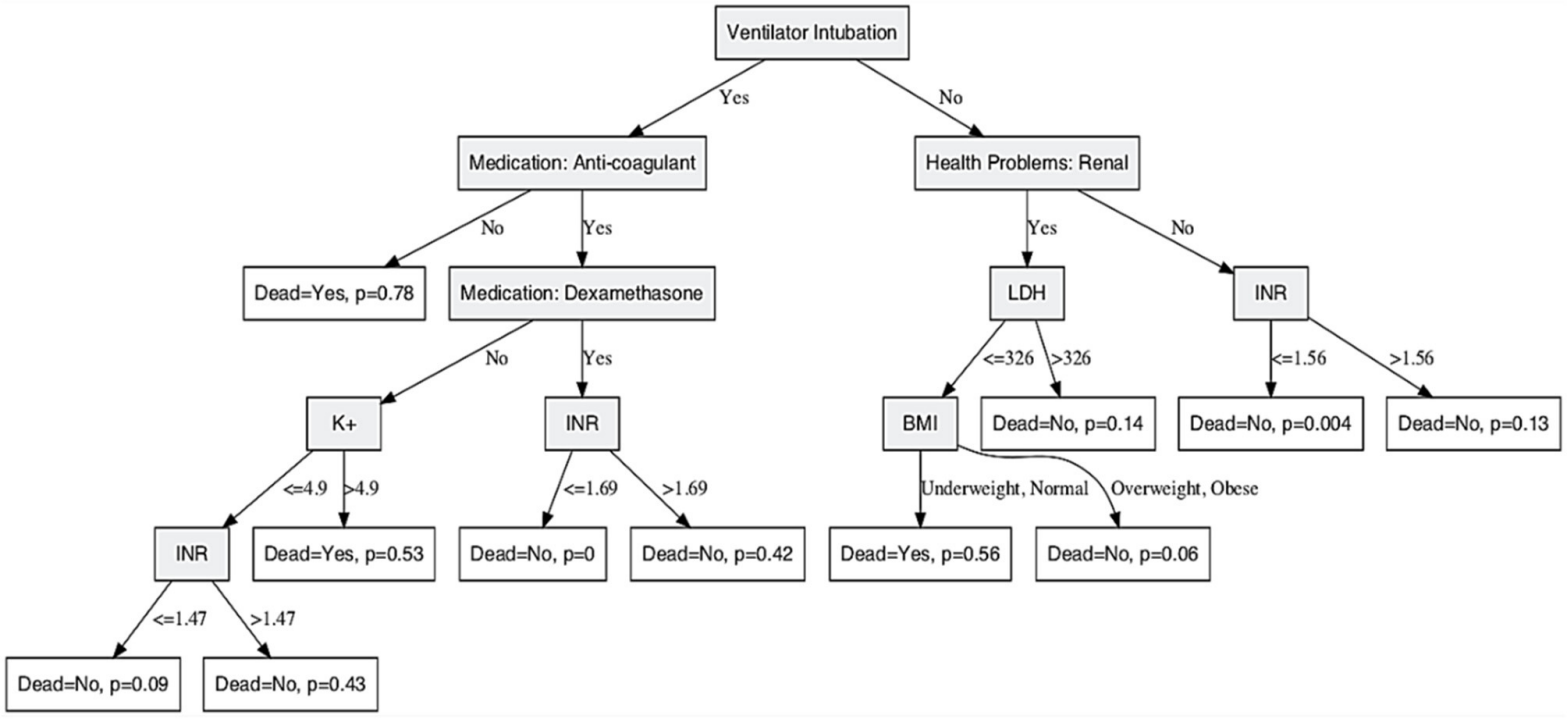
Decision tree model for predicting COVID-19 risk of death. A decision tree prediction model was constructed to predict patient's risk of death given demographic and clinical attributes at initial evaluation. Shaded nodes are the parent/split nodes, and the non-shaded nodes are the leaf nodes of the DT model. The conditional recursive partitioning tree algorithm (ctree) was used for model building. The parameter *p* shown at the leaf nodes is the risk of death for patients satisfying the conditions of that tree branch.

Death is the most severe consequence of SARS-CoV2 infection. According to the WHO, there are more than 14 million reported cases worldwide with more than 600,000 deaths. Therefore, it is of most importance to identify patients at a high risk of progression to severe disease and death. The identification of factors that contribute to the risk of death can help physicians to make appropriate decisions to reduce disease severity leading to death. A DT-based model was constructed to predict the risk of death based on patients' clinical and non-clinical information available at initial evaluation. The constructed model shown in Figure 4 was trained and validated on 75% of all patients' records, and its performance was evaluated on the remaining 25% of patients' records.

Performance evaluation results show an overall prediction accuracy of 96% with model sensitivity and specificity of 96.5 and 87.8%, respectively. Significant predictors of risk of death were intubation; BMI; presence of renal health problems; medications: anti-coagulant, dexamethasone; and lab test results: INR, LDH, and K+. A high risk of death of 78% is indicated in Figure 4 for intubated patients who have not used anti-coagulant medications. Intubated patients who are using anticoagulants and dexamethasone, and their INR is <1.69 have zero risk of death. Patients who are not intubated, have no kidney-related problems, and their INR is <1.56 have a very low risk of death of 0.4%. However, intubated patients who are using anticoagulants but not dexamethasone have a risk of death of 53% if their potassium level is more than 4.9 mmol/L.

**Figure 5:**
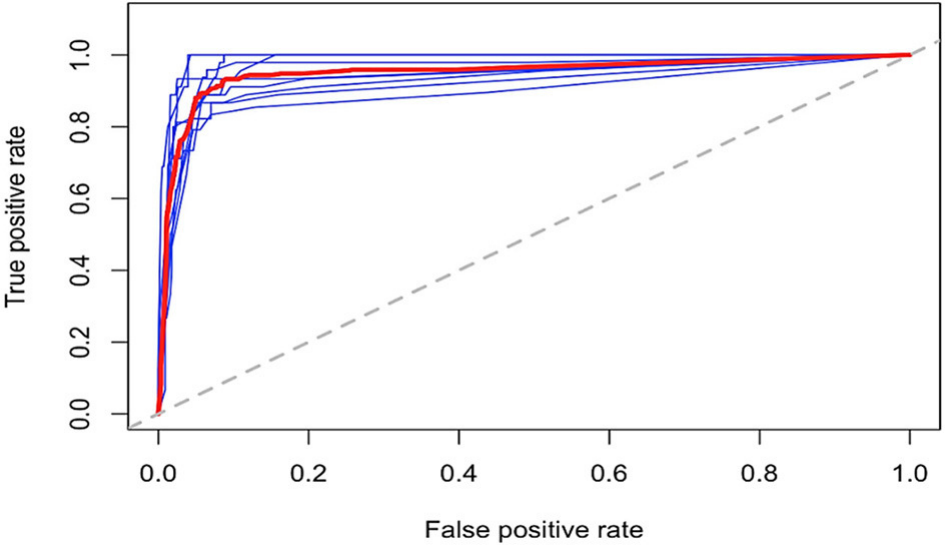
Risk of death ROC curves for the tenfold cross-validation subsets. ROC curves for the tenfold training data used at the model-building step. Blue lines represent the ROC curves at each cross-validation fold, and the red bold line represent the average across all cross-validation folds.

Variable importance for predicting risk of death was estimated through a random forest technique and is shown in Supplementary Figure 5. The top three most important variables were the use of a ventilator, anticoagulant drugs, and bronchodilator drugs, respectively. The variables use of a ventilator and anticoagulant drugs were identified by our provided DT model in Figure 4 as important variables in predicting risk of death. The use of bronchodilator drugs was not used in the model probably due to its high correlation with other surrogate variables. Model performance for the different subsets used in the tenfold cross-validation model performance evaluation step is shown through the receiver operator characteristic (ROC) curves in Figure 5. Curves shown in Figure 5 are very similar to the overall average ROC curve across all folds indicating a stable and robust DT model performance.

## Discussion

The COVID-19 crisis is a healthcare priority with significant sociological and economic impact globally. Almost all nations, many private and public organizations, and individuals are exploring different angles to handle the COVID-19 outbreak as it continues. Data analytics is one of the approaches that is widely used in such exploration. Early diagnostic and predictive modeling can enhance the therapeutic options available, leading to improvements in the overall clinical outcomes, and enable the allocation of limited resources to tackle COVID-19 more effectively. Epidemiologically, more than 14 million confirmed COVID-19 cases and more than 600,000 deaths are reported worldwide. Thus, minimizing the risk of death and providing an analytical tool to accurately predict the risk of death is crucial. In this study, we used multivariate analysis to identify the key variables and then used those to build prediction modeling using the DT algorithm to analyze and develop predictive models based on COVID-19 patient data. Analysis of medical records of 2017 COVID-19 positive patients who were mostly middle-aged overweight or obese men of Asian ethnicity showed that obesity-derived inflammation among middle-aged men results in an immunological disadvantage against SARS-CoV2 infection. Indeed, the analysis suggests that advancing age and higher BMI levels showed a positive association with disease severity, which is consistent with previous publications (13–15). Surprisingly, about 94% of the COVID-19 patients in our study had a positive Rhesus factor (Rh) blood group, whereas a previous study reported a lower level of Rh-positive blood type among COVID-19 positive patients, ~40% (16). Perhaps Rh-positive blood type may implicate a unique genetic susceptibility to acquiring SARS-CoV2 infection, such a mechanism is yet to be elucidated.

The two DT-based predictive models were constructed to provide rapid clinical decision-making support. Both models were built and trained using all of the existing data of COVID-19 patients to predict with high, 96%, accuracy the LOS and risk of death. Hospital LOS effective management while under the COVID-19 pandemic will save lives and is currently regarded as a key resource indicator in many COVID-19 hard-hit countries (17). We constructed and trained a DT-LOS prediction based on significant attributes that were generated autonomously based on DT algorithms and resulted in diagnosis at admission; use of ventilator intubation; medications: painkillers, azithromycin, antiviral, anti-inflammatory, and vitamin C; and lab tests: urea, PLT, dimer, potassium, and hemoglobin level were used as model inputs. This model shows very good performance with a coefficient of determination *R*^2^ of 49.8% and a MAD of 3.9 days. Based on the obtained DT model, urea levels above or below 51 mg/dL seem to be an important indicator for LOS. A recent study revealed that acute kidney injury among patients with COVID-19 is a key indicator for in-hospital death, including elevated urea levels (18). Here, we show a dramatic reduction in LOS from 14.35 to 2.77 days based on the urea level in correlation with other attributes based on the DT-LOS model. For example, COVID-19 patients with urea levels more than 51 mg/dL and are not on ventilation but use painkillers, they stay for 14.35 days on average in hospital, whereas patients with urea levels <51 mg/dL, PLT level <297,000 platelets/μL, dimer level <1.03 mcg/mL, have no comorbidities and have not used antiviral or anti-inflammatory medications stay in the hospital for 2.77 days on average. Other important attributes were platelet count and dimer level; mounting evidence suggests that COVID-19 patients develop coagulation problems based on disease severity leading to disseminated intravascular coagulation and strokes. For example, a research group in Germany noted an elevated level of Dimer and mild thrombocytopenia in COVID-19 autopsy (19). Other studies also suggest a 20–30% increase in stroke among seriously ill COVID-19 patients (20, 21). Interestingly, we determined a significant association between platelet and dimer levels and disease severity as shown in Figure 1 and deemed key attributes in the DT-LOS model. Further, we revealed that ventilation use in COVID-19 patients may influence LOS ranging from 7.7 days with no ventilation use to 45.4 days if ventilation is used among other attributes in the DT-LOS model. A study reported a significant improvement in respiratory function after 6 weeks of respiratory rehabilitation in elderly patients with COVID-19 (22). Therefore, we recommend pursed-lip and diaphragmatic breathing exercises, especially for people who are at higher risk for severe COVID-19 illness (23, 24).

Finally, we extrapolated a DT-based model to predict the risk of death in COVID-19 patients, which can also be considered as an indirect measure of disease severity. Astoundingly, the model shows excellent performance with an overall prediction accuracy of 96%. Model sensitivity and specificity were 96.5 and 87.8%, respectively. Remarkably, we showed 78.2% risk of death for intubated patients who have not used anticoagulant medications as shown in Figure 4. Fortunately, intubated patients who are using anticoagulants and dexamethasone and their INR is <1.69 have zero risk of death. Recently, an ongoing clinical trial suggested a breakthrough in COVID-19 management using dexamethasone. A preprint article from the University of Oxford concludes that dexamethasone reduces mortality among COVID-19 patients who are receiving ventilation or oxygen without ventilation. Interestingly, the risk of death DT model shows an intriguing role for dexamethasone in saving lives, ranging from zero risk of death if used to 0.429 risk of death if not used among other attributes as mentioned above. Dexamethasone inhibits IL-1 and TNF activity in the lung fibroblasts and, thus, reduces lung fibrosis, a common COVID-19 complication (25). However, the exact mechanism of dexamethasone action in SARS-CoV-2 infection is yet to be elucidated. The DT model was further validated by unsupervised learning methods showing similar separation patterns, and the ROC approach suggests a stable and robust DT model performance. Despite these meticulous validation techniques, we acknowledge the need for further validation of the algorithm on patients from different geographical and ethnic backgrounds as a potential limitation of this study.

In conclusion, we reviewed 2017 COVID-19 infected patients' medical records and analyzed relevant demographic-, laboratory-, and management-related modalities to combat SARS-CoV-2 infection. We successfully identified patterns of data fluctuation based on the level of care provided that reflects COVID-19 severity. Then we identified, trained, and tested LOS and risk of death DT-based models with high accuracy. The wild spread of SARS-CoV-2 infection imposes a logistical problem and major challenge to healthcare systems. Therefore, it is hoped that our DT-based models will act as a clinical expert system to address this urgent need and facilitate more effective COVID-19 management strategies that can save the lives of COVID-19 infected patients.

## Data Availability Statement

The raw data supporting the conclusions of this article will be made available by the authors, without undue reservation.

## Ethics Statement

The Study Protocol was reviewed and approved by Dubai Scientific Research Ethical Committee (DSREC) Dubai Health Authority (DHA); the Ethical Approval Number of the study is DSREC-04/2020_11. Written informed consent for participation was not required for this study in accordance with the national legislation and the institutional requirements.

## Author Contributions

MA, HA, AS, and BM: conception and design. BM and LS: acquisition of data. BM, LS, and HA: processing of specimens and generation of data. BM, HA, AS, MA, RH, and LS: analysis, interpretation of data, drafting or revising of manuscript, and final approval of manuscript. MA: has access to all study data and takes responsibility for the data integrity and accuracy. All authors contributed to the article and approved the submitted version.

## Conflict of Interest

The authors declare that the research was conducted in the absence of any commercial or financial relationships that could be construed as a potential conflict of interest.

## Abbreviations

BMI: Body mass index
COVID-19: Coronavirus disease 2019
DT: Decision tree
LOS: length of stay
MEWS: Modified Early Warning Score
SARS-CoV-2: Severe acute respiratory syndrome coronavirus 2
WHO: World Health Organization.

## References

[1] Huang C Wang Y Li X Ren L Zhao J Hu Y. Clinical features of patients infected with 2019 novel coronavirus in Wuhan, China. Lancet. (2020) 395:497–506. 10.1016/S0140-6736(20)30183-5 31986264 PMC7159299

[2] Lai Y Yeung W Celi LA. Urban intelligence for pandemic response: viewpoint. JMIR Public Health Surveill. (2020) 6:e18873. 10.2196/18873 32248145 PMC7159057

[3] Silver D Schrittwieser J Simonyan K Antonoglou I Huang A Guez A. Mastering the game of Go without human knowledge. Nature. (2017) 550:354–9. 10.1038/nature24270 29052630

[4] Pickering BW Dong Y Ahmed A Giri J Kilickaya O Gupta A. The implementation of clinician designed, human-centered electronic medical record viewer in the intensive care unit: a pilot step-wedge cluster randomized trial. Int J Med Inform. (2015) 84:299–307. 10.1016/j.ijmedinf.2015.01.017 25683227

[5] Steele AJ Denaxas SC Shah AD Hemingway H Luscombe NM. Machine learning models in electronic health records can outperform conventional survival models for predicting patient mortality in coronary artery disease. PLoS ONE. (2018) 13:e0202344. 10.1371/journal.pone.0202344 30169498 PMC6118376

[6] Alimadadi A Aryal S Manandhar I Munroe PB Joe B Cheng X. Artificial intelligence and machine learning to fight COVID-19. Physiol Genom. (2020) 52:200–2. 10.1152/physiolgenomics.00029.2020 32216577 PMC7191426

[7] Vaishya R Javaid M Khan IH Haleem A. Artificial intelligence (AI) applications for COVID-19 pandemic. Diabetes Metab Syndr. (2020) 14:337–9. 10.1016/j.dsx.2020.04.012 32305024 PMC7195043

[8] Kumar A Gupta PK Srivastava A. A review of modern technologies for tackling COVID-19 pandemic. Diabetes Metab Syndr. (2020) 14:569–73. 10.1016/j.dsx.2020.05.008 32413821 PMC7204706

[9] Friedman J Hastie T Tibshirani R. The Elements of Statistical Learning. New York, NY: Springer Series in Statistics (2001).

[10] Hothorn T Hornik K Zeileis A. Unbiased recursive partitioning: a conditional inference framework. J Comput Graphical Stat. (2006) 15:651–74. 10.1198/106186006X133933

[11] Team RC. R version 3.5. 3: A Language and Environment for Statistical Computing. R Foundation for Statistical Computing, Vienna, Austria. (2019). Available online at: https://www.R-project.org (accessed May 5, 2020).

[12] Ho TK editor. Random decision forests. In: Proceedings of 3rd International Conference on Document Analysis and Recognition IEEE (Montreal, QC) (1995).

[13] Kuderer NM Choueiri TK Shah DP Shyr Y Rubinstein SM Rivera DR. Clinical impact of COVID-19 on patients with cancer (CCC19): a cohort study. Lancet. (2020) 395:1907–18. 10.1016/S0140-6736(20)31187-9 32473681 PMC7255743

[14] Cummings MJ Baldwin MR Abrams D Jacobson SD Meyer BJ Balough EM. Epidemiology, clinical course, and outcomes of critically ill adults with COVID-19 in New York City: a prospective cohort study. Lancet. (2020) 395:1763–70. 10.1016/S0140-6736(20)31189-2 32442528 PMC7237188

[15] Kass DA Duggal P Cingolani O. Obesity could shift severe COVID-19 disease to younger ages. Lancet. (2020) 395:1544–5. 10.1016/S0140-6736(20)31024-2 32380044 PMC7196905

[16] Zietz M Tatonetti NP. Testing the association between blood type and COVID-19 infection, intubation, and death. Nature Commun. (2020) 11:1–6. 10.1101/2020.04.08.20058073 33188185 PMC7666188

[17] Rees EM Nightingale ES Jafari Y Waterlow N Clifford S Pearson CAB. COVID-19 length of hospital stay: a systematic review and data synthesis. BMC Med. (2020) 18:1–22. 10.1186/s12916-020-01726-3 32878619 PMC7467845

[18] Cheng Y Luo R Wang K Zhang M Wang Z Dong L. Kidney disease is associated with in-hospital death of patients with COVID-19. Kidney Int. (2020) 97:829–38. 10.1016/j.kint.2020.03.005 32247631 PMC7110296

[19] Wichmann D Sperhake JP Lutgehetmann M Steurer S Edler C Heinemann A. Autopsy findings and venous thromboembolism in patients with COVID-19. Ann Internal Med. (2020) 173:1030. 10.7326/L20-1206 33316197

[20] Klok FA Kruip M van der Meer NJM Arbous MS Gommers D Kant KM. Incidence of thrombotic complications in critically ill ICU patients with COVID-19. Thromb Res. (2020) 191:145–7. 10.1016/j.thromres.2020.04.013 32291094 PMC7146714

[21] Poissy J Goutay J Caplan M Parmentier E Duburcq T Lassalle F. Pulmonary embolism in COVID-19 patients: awareness of an increased prevalence. Circulation. (2020) 142:184–6. 10.1161/CIRCULATIONAHA.120.047430 32330083

[22] Liu K Zhang W Yang Y Zhang J Li Y Chen Y. Respiratory rehabilitation in elderly patients with COVID-19: a randomized controlled study. Complement Ther Clin Pract. (2020) 39:101166. 10.1016/j.ctcp.2020.101166 32379637 PMC7118596

[23] Mendes LP Moraes KS Hoffman M Vieira DS Ribeiro-Samora GA Lage SM. Effects of diaphragmatic breathing with and without pursed-lips breathing in subjects with COPD. Respir Care. (2019) 64:136–44. 10.4187/respcare.06319 30154127

[24] Ubolnuar N Tantisuwat A Thaveeratitham P Lertmaharit S Kruapanich C Mathiyakom W. Effects of breathing exercises in patients with chronic obstructive pulmonary disease: systematic review and meta-analysis. Ann Rehabil Med. (2019) 43:509–23. 10.5535/arm.2019.43.4.509 31499605 PMC6734022

[25] Monick MM Aksamit TR Geist LJ Hunninghake GW. Dexamethasone inhibits IL-1 and TNF activity in human lung fibroblasts without affecting IL-1 or TNF receptors. Am J Physiol. (1994) 267:L33–8. 10.1152/ajplung.1994.267.1.L33 7519403

