# Supplementary material for: Prediction of COVID-19 Hospital Length of Stay and Risk of Death Using Artificial Intelligence-Based Modeling
Source: Front Med (Lausanne). 2021 May 4;8:592336. doi: 10.3389/fmed.2021.592336 (PMC8129500; doi:10.3389/fmed.2021.592336)
Supplement: Supplementary file 1 [file Data_Sheet_1.PDF]

Prediction of COVID-19 length of stay and risk of death using artificial intelligence-based modeling.

Bassam Mahboub, MD[1], Mohammad T. AL Bataineh, MD[1,2]*, Hussam Alshraideh, PhD[3,4]*, Rifat Hamoudi, PhD[1,2,5], Abdulrahim Shamayleh, PhD[3], Laila Salameh, MS[2]

Supplementary material

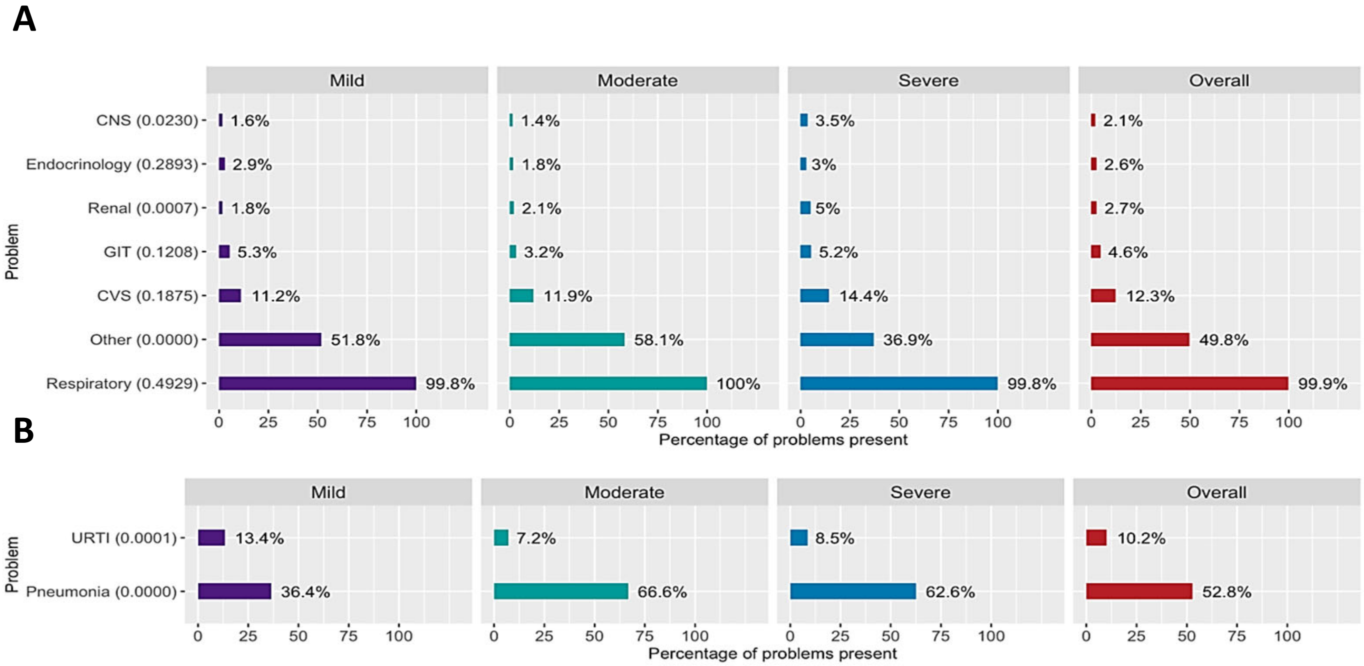

Figure S1. Percentage of health problems present at admission. Based on MEWS scores, overall health problems were categories into three levels according to their COVID-19 disease severity and percentage of each health problem among the three categories was estimated as the number of patients with the reported health problem over the total number of patients in that category (A). Respiratory health problem was further categorized into URTI and pneumonia (B). Numbers in parentheses are the Chi-squared p-values for testing for significant differences among severity levels.

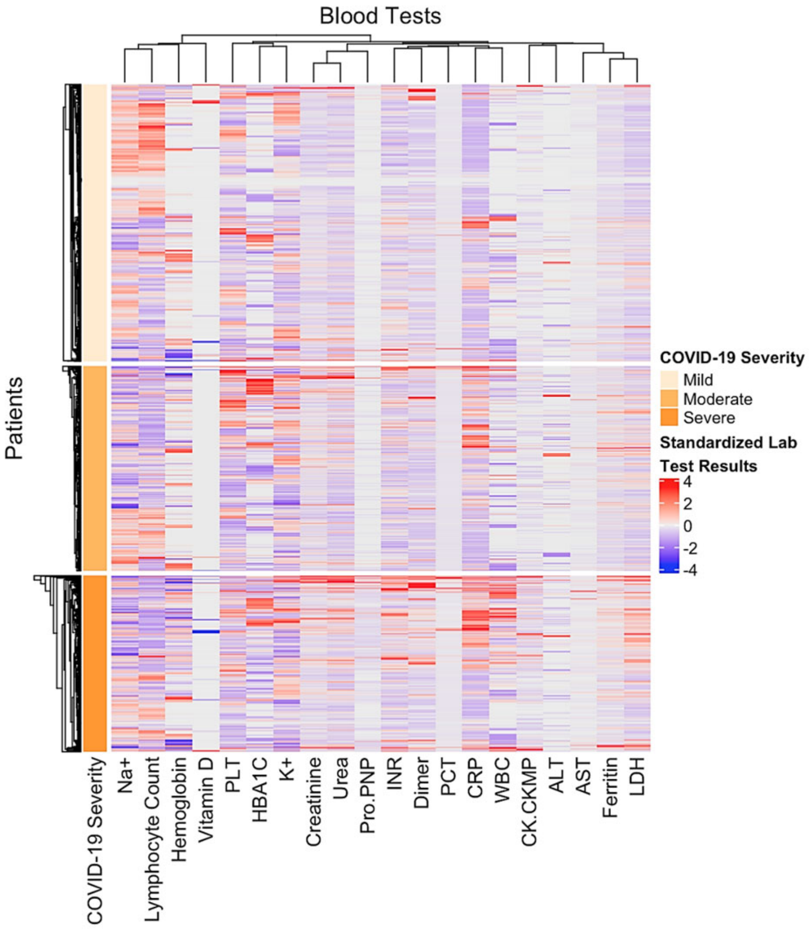

Figure S2. Heatmap of patients' standardized lab test results according to their disease severity levels. Unsupervised clustering of patient's (rows) and blood tests (columns) was constructed using Euclidean distance measure and Ward linkage. Data was first standardized by subtracting the mean and dividing by the standard deviation for each lab test. Patients clusters are further divided by the severity level.

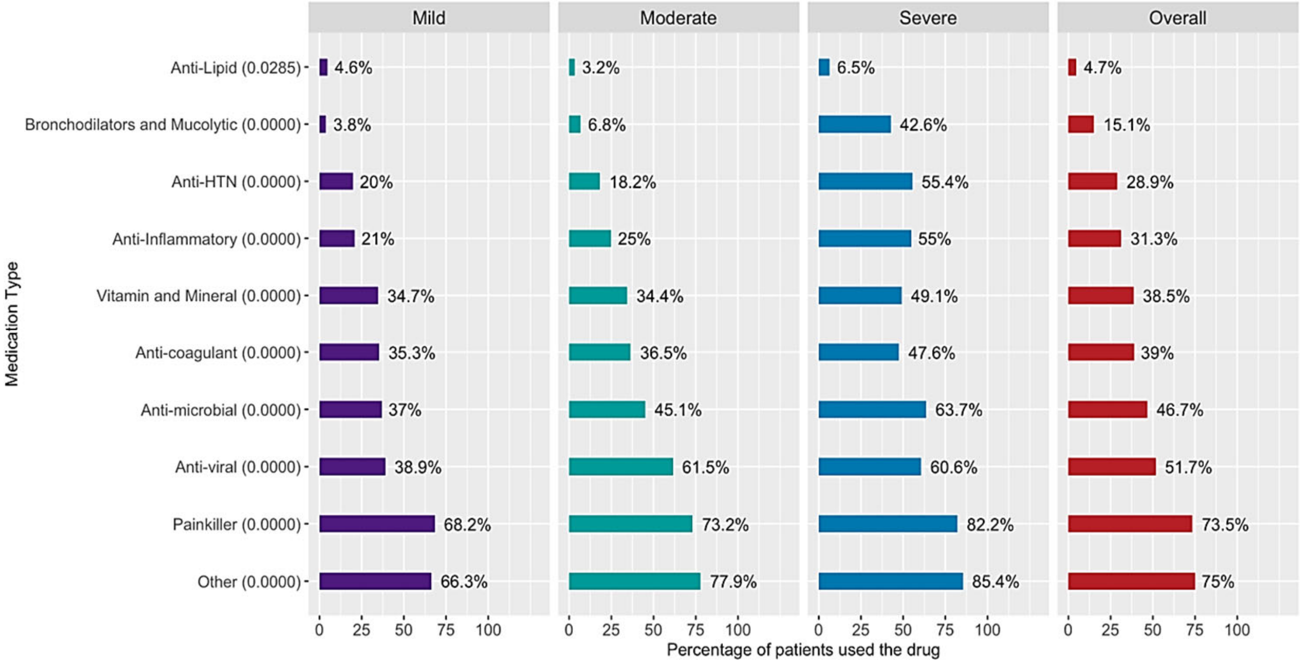

Figure S3. Percentage of patients with all medications prescribed. Percentage of patients prescribed medications shown in the y-axis of the figure. Proportions were calculated as the proportion of patients prescribed the medication of all patients at that severity level. Numbers in parentheses are the Chi-squared p-values for testing for significant differences among severity levels.

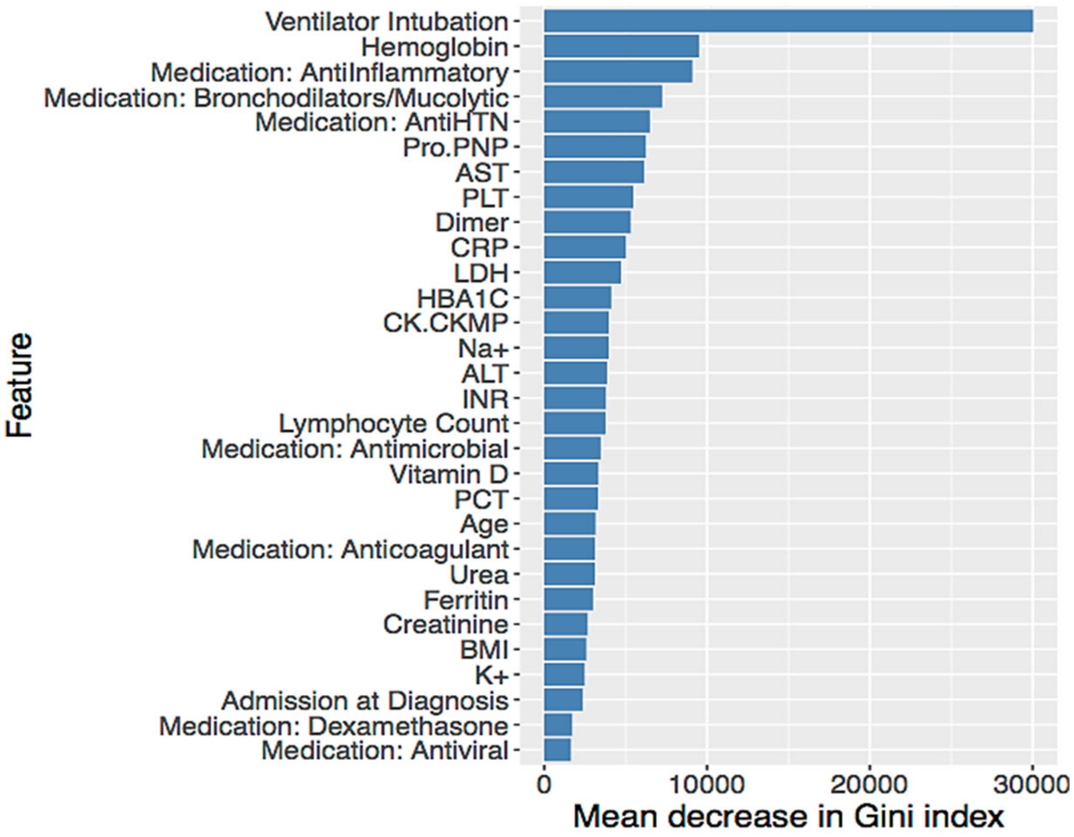

Figure S4. Variable importance for predicting length of stay. Variable importance for predicting length of stay was estimated through random forest ensembling technique. Node impurity was assessed through the Gini index and the top 30 variables are shown only for display purpose.

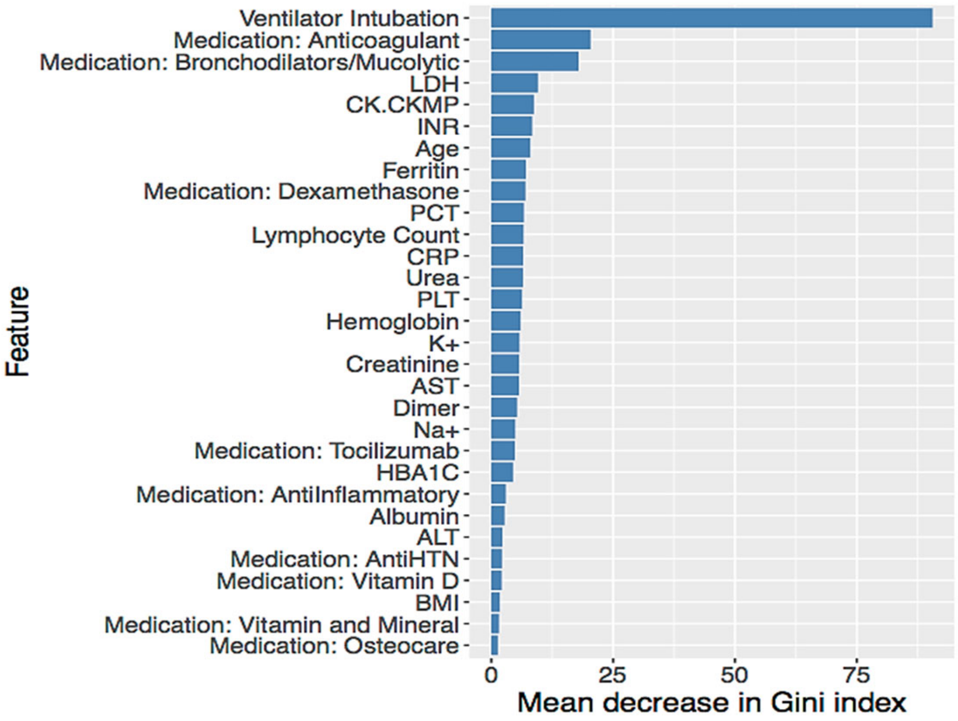

Figure S5. Variable importance for predicting risk of death. Variable importance for predicting risk of death was estimated through random forest ensembling technique. Node impurity was assessed through the Gini index and the top 30 variables are shown only for display purpose.
